# Elevated blood urea nitrogen-to-creatinine ratio increased the risk of Coronary Artery Disease in patients living with type 2 diabetes mellitus

**DOI:** 10.1186/s12902-022-00954-3

**Published:** 2022-02-28

**Authors:** Feng Liu, Guanhui Ma, Chao Tong, Shan Zhang, Xinghua Yang, Cong Xu, Weihao Yang, Guobao Xia, Mingliang Li

**Affiliations:** 1 Center for Quality Control and Improvement of Physical Examination, Beijing Physical Examination Center, No. 1, Yard 81, Fucheng Road, Beijing, Haidian District China; 2 Research Center of Digital Health China, Health and Medical Research Institute, Jinan, China; 3 Department of Big Data, Kangping Medical Health Co. Ltd, Jinan, China; 4 School of Public Health, Capital Medical University, No.10 Xitoutiao, Youanmen, Beijing, 100069 China

**Keywords:** UCR, T2DM, CAD, Cox regression, Cubic spline function, Kaplan–Meier survival

## Abstract

**Background:**

High Blood Urea Nitrogen (BUN) and high Serum Creatinine (SCr) levels are risk factors for Coronary Artery Disease (CAD). However, the relationship between the Blood Urea Nitrogen to Creatinine (BUN/SCr) ratio (UCR) and the risk of CAD in patients living with new-onset diabetes is unclear. This study aimed to examine the relationship between blood UCR and the risk of CAD in patients living with new-onset type 2 diabetes mellitus (T2DM).

**Methods:**

We analyzed the data from the cohort of 12,299 patients living with type 2 diabetes mellitus. Primary endpoints were the events of CAD. The ANOVA test (continuous indicators) and χ^2^ test (categorical indicators) were used to assess the differences of baseline characteristics across the groups of UCR. In order to understand the correlation between variables, we performed correlation analysis on variables that have significant differences between CAD group and non-CAD group. Multivariate-adjusted Cox proportional hazard regression models were applied to estimate the association of the blood UCR with the risk of CAD in patients living with T2DM. The Kaplan–Meier survival function plotting and the log-rank test were used to evaluate the event-free survival according to the groups of UCR. The restricted cubic spline model was used to show the adjusted association between blood UCR and risk of CAD in patients living with T2DM.

**Results:**

During a median follow-up of 2.66 years, 1173 CAD were recorded with an event rate of 28.49 events per 1000 person-years. In multivariate-adjusted Cox regression models, elevated blood urea nitrogen to creatinine ratio (UCR) was associated with higher risk of CAD in patients living with T2DM [hazard ratio (HR), 1.782; 95% confidence interval (CI), 1.237–2.567]. The Kaplan–Meier survival curves indicated that the high group of UCR tended to have a lower event-free survival than the low group and medium group. There was a nonlinear trend toward increasing risk of CAD across the groups of UCR. And cubic spline function graph suggested that the influence of UCR level on HR for CAD increased significantly at UCR levels above 6.67.

**Conclusions:**

An elevated UCR was significantly associated with an increased risk for CAD in patients living with T2DM.

## Background

In recent years, owing to the environmental factors such as sedentary lifestyles and changing dietary habits, type 2 diabetes mellitus (T2DM) has gradually become a global health problem [1]. According to the International Diabetes Federation (IDF) report, the age-standardized prevalence rate of diabetes in 2019 is 8.3%, which is expected to reach 9.6% in 2045 [2]. In China, T2DM has imposed a heavy economic burden on patients and society, and the total economic burden of T2DM and its complications reached 247.8 billion RMB in 2007 [3]. If patients living with T2DM do not take an active role in effectively controlling and treating their diseases, they are prone to a variety of diabetes-related complications that are responsible for the impaired quality of life, disability, and premature death. CAD (Coronary Artery Disease) is a cardiovascular complication of DM [4]. In a systematic evaluation of 4,549,481 patients living with T2DM, the total incidence of macrovascular complications was 32.2%, and CAD was one of the most common cardiovascular diseases (21.2%) [5].

The dysfunction of kidney not only implicates potential pathological changes of the kidney, but could also influence other organs and systems as a result of the impairments of body homoeostasis [6]. Cardiovascular system is one of the most affected systems upon the impairment of renal function [7]. Blood urea nitrogen (BUN), a metabolic product of protein, is susceptible to external factors and is a very sensitive indicator of changes in haemodynamic and renal perfusion. Studies have shown that BUN is strongly associated with mortality in patients with heart failure [8–12]. Haijing Jiang et.al found that a raised level of BUN might be associated with increased risk of incident CAD in Chinese populations [13]. Serum creatinine (SCr) is mainly determined by the glomerular filtration capacity. Previous meta-analysis has observed that an impaired renal function, defined as estimated glomerular filtration rate(eGFR) lower than 60 mL/min per 1.73 m^2^, was associated with an increased risk of incident CAD, independently of prevalent hypertension and diabetes [14]. Emanuele Di Angelantonio et.al found that there was a moderate increase in CAD risk associated with very low eGFR in the general population [15]. Furthermore, recently, the blood urea nitrogen to creatinine ratio (UCR) has emerged as an independent predictor of adverse clinical outcomes in various population settings, such as acute kidney injury [16], chronic heart failure [17–19], and ischemic stroke [20]. However, epidemiological evidence that related UCR to risk of incident CAD in patients living with T2DM was still limited.

In the present study, based on a cohort of health management population from Beijing Physical Examination Centre, we investigated the association of UCR with risk of incident CAD, and hope to help improve clinical control of CAD among T2DM patients.

## Methods

### Study population

Through cooperation with Beijing Municipal Health Commission Information Centre and Beijing Centre for Diseases Prevention and Control, Beijing Physical Examination Centre has successfully realized the interconnection of physical examination data, electronic medical records data, and cause of death data.

The prospective cohort study was conducted on the medical examination data obtained in Beijing Physical Examination Centre from January 2009 through December 2019. New-onset T2DM participants aged between 20 to 90 without CAD, stroke, and cancer were included in the cohort study. Physical examination records before January 2009 were excluded which contained 40,841 records. Non-new-onset diabetes and participants diagnosed with CAD, stroke, AF, cancer before the onset of diabetes were excluded which contained 25,304 persons. In addition, Patients with lack of blood routine indicators (*White Blood Cell Count (WBC), Neutrophil Count (NEUT), Lymphocyte Count (LYM)*), blood biochemical indicators (*Fasting Plasma Glucose (FPG), Fasting Plasma Glucose Followed (FPG_F), Total Cholesterol (TC), Triglyceride (TG), High-Density Lipoprotein Cholesterol (HDL-C), Low-Density Lipoprotein Cholesterol (LDL-C), BUN, SCr* and general inspections (*Body Mass Index (BMI), Waist-To-Hip Ratio (WHR), Systolic Blood Pressure (SBP), Diastolic Blood Pressure (DBP)*), or the absence of a follow up visit, or outliers, which contained 12,114 persons, were excluded in the final cohort of this study.

As there are no references for outlier ranges of the indicators for the health management population, we use the outlier ranges of the diseased population indicators defined by authoritative epidemiologist to replace, which is the limitation of this study. The outliers range of BMI are less than 10kg/m^2^ or greater than 50kg/m^2^. The outliers of SBP and DBP are less than 60 mmHg or greater than 250 mmHg, less than 30 mmHg or greater than 150 mmHg respectively. The outliers of FPG are less than 1.0 mmol/L or greater than 30.0 mmol/L. The outliers of TG are less than 0.1 mmol/L or greater than 30.0 mmol/L. The outliers of TC are less than 1.0 mmol/L or greater than 15.0 mmol/L. The outliers of HDL-C are greater than 10.0 mmol/L. The outliers of LDL-C are greater than 10.0 mmol/L. The outliers of BUN are greater than 30.0 mmol/L. The outliers of SCr are less than 15umol/L or greater than 4500umol/L. The outliers of WBC are greater than 100 109/L. The outliers of NEUT are greater than 30 109/L. The outliers of LYM are greater than 30 109/L.

#### Clinical measurements

The medical examination data combines a questionnaire survey, general inspections, routine blood index test, biochemical index test, and so on. The content of the questionnaire mainly included age, sex, medical history, and other basic information. *Systolic Blood Pressure (SBP)* and *Diastolic Blood Pressure (DBP)* were measured twice within 5 min, took the average value of the two results, and recorded it as the final value of *Systolic Blood Pressure (SBP)* and *Diastolic Blood Pressure (DBP)*. The height and weight of participants were measured by standard electronic equipment and recorded. According to the regulations of World Health Organization, *Body Mass Index (BMI)* was calculated as weight (*kg*) divided by height squared (*m*^*2*^). Waist circumference and hip circumference were measured by professionally trained medical staff with soft ruler. Before the biochemical index test, all participants were required to fast for at least 10 h to measure *Fasting Plasma Glucose (FPG)*, *Glycosylated Haemoglobin A1c (HbA1c), Total Cholesterol (TC)*, *Triglyceride (TG), High-Density Lipoprotein (HDL-C), Low-Density Lipoprotein (LDL-C), Blood Urea Nitrogen (BUN), Uric Acid (UA), Serum Creatinine (SCr), White Blood Cell Count (WBC), Neutrophil Count (NEUT), Lymphocyte Count (LYM)*, and so on.

This study was approved by the Ethics Committee of the Beijing Physical Examination Centre. All participants provided written informed consent. All the methods in the present study were carried out following the ethical guidelines of the 1975 Declaration of Helsinki.

### Diagnostic criteria of participants

#### Type 2 diabetes mellitus (T2DM)

According to the Guideline for the prevention and treatment of type 2 diabetes mellitus in China, T2DM was diagnosed if it meets one of the following criteria: ①with typical symptoms of diabetes (polydipsia, polyuria, polyphagia, decreased body mass), and a random blood glucose level ≥ 11.1 mmol/L; ②fasting blood glucose ≥ 7.0 mmol/L, or plasma glucose of 2 h post glucose load ≥ 11.1 mmol/L, or HbA1c ≥ 6.5%; ③a previous medical diagnosis [21, 22].

In this study, the diagnostic of T2DM comprehensively considered FPG, 2 h postprandial blood glucose, HbA1c and T2DM records. Values of FPG, 2 h postprandial blood glucose, and HbA1c were obtained from medical examination data. T2DM records were obtained from questionnaire data, electronic medical records data (only E11.0, E11.1, E11.2–E11.9(ICD-10) were found in our dataset) [23], and cause of death data. The earliest diagnosis of diabetes was used as the onset date.

#### Coronary Artery Disease (CAD)

The individuals who were diagnosed with CAD at the Beijing Physical Examination Centre underwent electrocardiographic (ECG) examination and angiography. Individuals with abnormal ECG like angina pectoris, myocardial infarction or myocardial ischaemia were diagnosed with CAD. Besides, we also referred to the CAD records in the cause of death data (I20-25 from ICD10 Codes) [24, 25] and the electronic medical records. The earliest diagnosis of CAD was used as the onset date.

### Statistical analysis

Data was presented as means ± standard deviations for continuous variables and as n(%) for categorical ones. In this study, according to the method of the normal value range in statistics, we used 5 and 95% quantiles to divide blood UCR into three groups. The 5 and 95% quantiles of UCR are 4.36 and 10.45 respectively. The differences of baseline characteristics across the groups of UCR were assessed using the ANOVA test for continuous indicators, and χ^2^ test for categorical ones [18]. Multivariate-adjusted Cox proportional hazard regression models were applied to estimate the association of the UCR with the risk of CAD in patients living with T2DM [26]. The model 1 was adjusted for age. The model 2 was adjusted for age, BMI, and DBP. The model 3 was adjusted for HDL-C, eGFR, and Triglyceride glucose (TyG) index plus the variables in the model 2. We calculated eGFR using equation of Chronic Kidney Disease Epidemiology Collaboration (CKD-EPI) [27]. And we used fasting triglyceride glucose (TyG) index to evaluate insulin resistance, which was calculated as ln[fasting triglycerides × fasting plasma glucose/2] [28]. The Kaplan–Meier survival function plotting and the log-rank test were used to evaluate the event-free survival according to the groups of UCR. The restricted cubic spline model fitted for Cox proportional hazards models was used to show the adjusted association between blood UCR and risk of CAD in patients living with T2DM [29].

All data analysis for this study was conducted using R software (version 3.6.3; https://www.R-project.org), and a two-tailed *P*-value of < 0.05 was regarded as statistically significant.

## Results

### Baseline characteristics

**Fig. 1:**
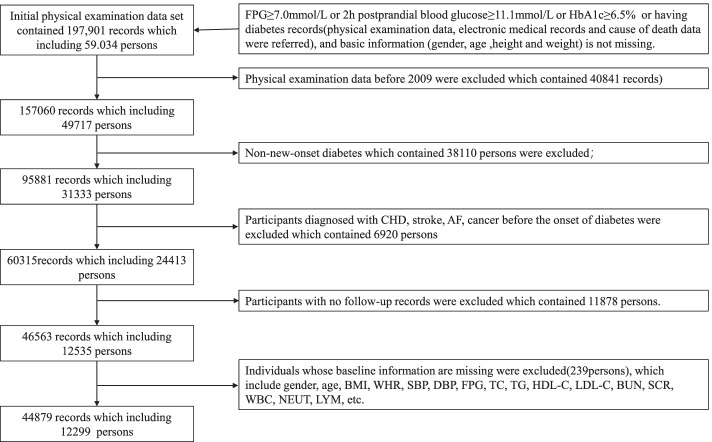
Flow chart of the cohort

At baseline, 12,299 participants (3860 women and 8439 men) with T2DM were enrolled into the cohort, and the flow chart of the cohort is shown in Fig. 1. The mean age was 52.53 ± 11.62 years for men and 52.67 ± 13.28 years for women. The overall mean ± standard deviation of blood UCR level was 6.92 ± 1.92.

Baseline characteristic of all participants divided by UCR levels in the cohort are shown in Table 1. Participants with a higher blood UCR were older and more likely to be women. The mean values of BMI, WAIST, WHR, SBP, DBP, WBC, NEUT, TG, eGFR and TyG decreased with increasing of UCR levels. In contrast, significant upward trends were observed in mean values for FPG, FPG_F, LDL-C, and HDL-C with increasing UCR levels. In addition, there are no significant differences in LYM and TC between different UCR levels.

**Table 1:** Baseline characteristics of participants in each group divided by UCR levels

Variables	All participants (*n* = 12,299)	Categories of blood Urea Nitrogen to Creatinine Ratio (UCR) level			
		Low level ≤ 4.36 (*n* = 614)	medium level 4.36–10.45 (*n* = 11,067)	High level > 10.45 (*n* = 618)	*P-value*
sex(men)	8,439 (68.62)	554 (90.23)	7663 (69.24)	222 (35.92)	< 0.001
age	52.57 ± 12.16	48.91 ± 11.19	52.65 ± 12.16	54.81 ± 12.31	< 0.001
Body Mass Index (BMI)	26.32 ± 3.7	27.02 ± 3.64	26.31 ± 3.69	25.94 ± 3.78	< 0.001
WAIST	88.93 ± 10.43	92.19 ± 10.48	88.92 ± 10.4	86 ± 10.07	< 0.001
Waist-To-Hip Ratio (WHR)	0.88 ± 0.06	0.9 ± 0.06	0.88 ± 0.06	0.86 ± 0.06	< 0.001
Systolic Blood Pressure (SBP)	129.84 ± 16.87	131.87 ± 16.54	129.86 ± 16.86	127.49 ± 17.07	< 0.001
Diastolic Blood Pressure (DBP)	83.42 ± 11.37	85.9 ± 11.83	83.46 ± 11.34	80.29 ± 10.8	< 0.001
White Blood Cell Count (WBC)	6.79 ± 1.73	7.03 ± 1.8	6.78 ± 1.72	6.7 ± 1.72	0.001
Neutrophil Count(NEUT)	3.98 ± 1.3	4.13 ± 1.42	3.97 ± 1.29	3.95 ± 1.3	0.016
Lymphocyte Count(LYM)	2.25 ± 0.73	2.29 ± 0.67	2.24 ± 0.73	2.23 ± 0.66	0.188
Fasting Plasma Glucose (FPG)	7.15 ± 2.32	6.98 ± 2.19	7.11 ± 2.26	7.93 ± 3.16	< 0.001
Fasting Plasma Glucose Followed (FPG_F)	7.05 ± 2.4	6.9 ± 2.27	7.01 ± 2.36	7.75 ± 3.05	< 0.001
Total Cholesterol (TC)	5.11 ± 1.01	5.11 ± 1.02	5.11 ± 1	5.2 ± 1.07	0.095
Low-Density Lipoprotein Cholesterol (LDL-C)	2.98 ± 0.86	2.84 ± 0.85	2.99 ± 0.85	2.97 ± 0.89	0.012
High-Density Lipoprotein Cholesterol (HDL-C)	1.19 ± 0.29	1.08 ± 0.26	1.2 ± 0.29	1.29 ± 0.31	< 0.001
Triglyceride (TG)	2.21 ± 2.39	3.34 ± 4.11	2.16 ± 2.21	2.08 ± 2.79	< 0.001
Estimated Glomerular Filtration Rate (eGFR)	105.05 ± 23.72	111.32 ± 29.66	105.13 ± 23.68	97.35 ± 13.64	< 0.001
Triglyceride glucose (TyG)	2.45 ± 0.8	2.71 ± 0.95	2.43 ± 0.79	2.41 ± 0.86	< 0.001

Values were expressed as mean ± standard deviation or n (%)

In order to explore the relationship between variables and CAD initially, baseline characteristics in each group divided by outcome was presented in Table 2. Participants with CAD were older and higher UCR level. The mean values of BMI, WAIST, WHR, SBP, DBP, FPG, LDL-C, eGFR, and TyG with CAD are significantly higher than those with non-CAD. The mean values of LYM and HDL-C with CAD are significantly lower than those with non-CAD. There are no significant differences in sex, WBC, NEUT, FPG_F, TC, and TG between CAD and non-CAD groups.

**Table 2:** Baseline characteristics of participants in each group divided by outcome

Variables	Total (*n* = 12,299)	without incident CAD (*n* = 11,126)	with incident CAD (*n* = 1173)	*P-value*
UCR_GROUP	2 ± 0.32	2 ± 0.32	2.03 ± 0.33	0.004
sex(men)	8,439 (68.62)	7624 (68.52)	815 (69.48)	0.524
age	52.57 ± 12.16	52.07 ± 12.21	57.37 ± 10.59	< 0.001
Body Mass Index (BMI)	26.32 ± 3.7	26.3 ± 3.72	26.53 ± 3.48	0.048
Waist	88.93 ± 10.43	88.84 ± 10.49	89.86 ± 9.78	0.001
Waist-To-Hip Ratio (WHR)	0.88 ± 0.06	0.88 ± 0.06	0.89 ± 0.06	< 0.001
Systolic Blood Pressure (SBP)	129.84 ± 6.87	129.55 ± 16.86	132.63 ± 16.68	< 0.001
Diastolic Blood Pressure (DBP)	83.42 ± 11.37	83.3 ± 11.43	84.58 ± 10.69	< 0.001
White Blood Cell Count (WBC)	6.79 ± 1.73	6.79 ± 1.74	6.75 ± 1.57	0.382
Neutrophil Count (NEUT)	3.98 ± 1.3	3.98 ± 1.31	3.95 ± 1.2	0.468
Lymphocyte Count (LYM)	2.25 ± 0.73	2.25 ± 0.74	2.21 ± 0.64	0.047
Fasting Plasma Glucose (FPG)	7.15 ± 2.32	7.16 ± 2.33	7.02 ± 2.23	0.04
Fasting Plasma Glucose Follow (FPG_F)	7.05 ± 2.4	7.05 ± 2.4	7.02 ± 2.34	0.712
Total Cholesterol (TC)	5.11 ± 1.01	5.11 ± 1.01	5.16 ± 1.01	0.098
Low-Density Lipoprotein Cholesterol (LDL-C)	2.98 ± 0.86	2.98 ± 0.85	3.03 ± 0.88	0.042
High-Density Lipoprotein Cholesterol (HDL-C)	1.19 ± 0.29	1.2 ± 0.29	1.16 ± 0.28	< 0.001
Triglyceride (TG)	2.21 ± 2.39	2.21 ± 2.41	2.26 ± 2.17	0.444
Estimated Glomerular Filtration Rate (eGFR)	105.05 ± 23.72	104.87 ± 23.37	106.67 ± 26.81	0.013
Triglyceride glucose (TyG)	2.51 ± 0.8	2.44 ± 0.81	2.58 ± 0.77	0.015

Values were expressed as mean ± standard deviation or n (%)

Abbreviations: *UCR* urea nitrogen to creatinine ratio

#### Clinical outcomes

**Fig. 2:**
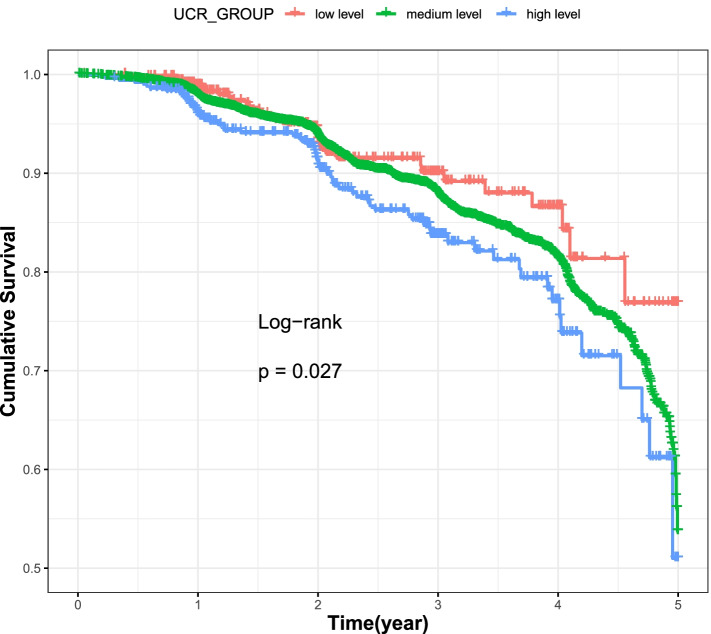
The Kaplan–Meier curves of the cumulative event-free survival rate according to the three levels of blood UCR

The median follow-up period was 2.66 years (range, 0–10.79 years), and 1173 CAD were recorded as having CAD for T2DM over 41,178.83 person-years follow-up, which yielded an event rate of 28.49 events per 1000 person-years. Of those, 358 CAD occurred in women and 815 CAD occurred in men. Kaplan–Meier survival curves were represented in Fig. 2, indicating that those at the high-level group of UCR tended to have a lower event-free survival than at the low level and the medium level groups (${\chi }^{2}$=13.1; log-rank *P* = 0.027, df = 2).

### Collinearity diagnostics and risk of CAD in different UCR levels

#### Collinearity diagnostics

**Fig. 3:**
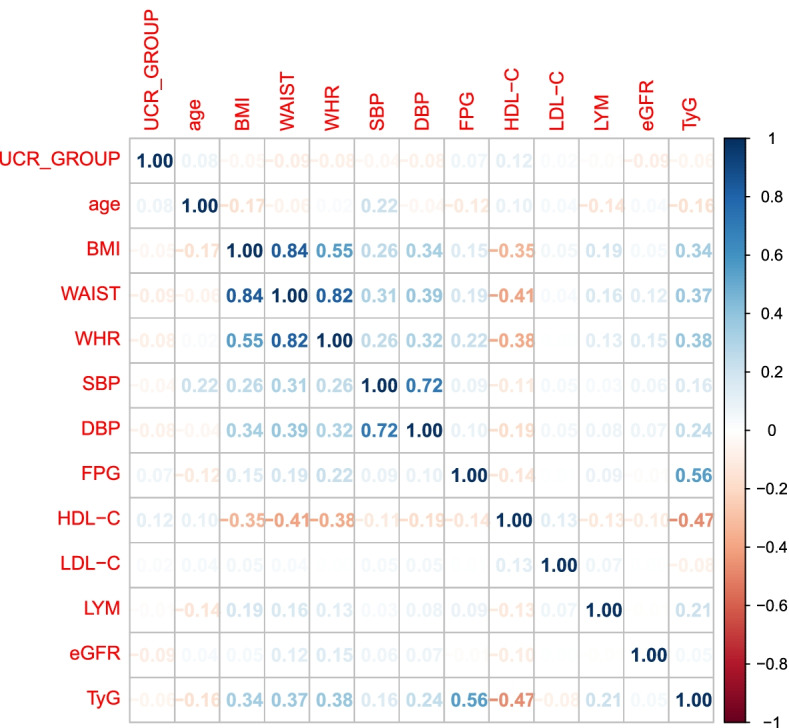
The correlation between variables

In order to understand the correlation between variables, we performed correlation analysis on variables that have significant differences between CAD group and non-CAD group. The correlation analysis diagram was shown in Fig. 3. We can see BMI, WHR, and WAIST have significant correlations, SBP and DBP have a significant correlation, and FPG and TyG have a significant correlation.

#### Risk of CAD in different UCR levels

In this study, we use stepwise regression to solve linear correlation problems. Multivariable Cox proportional hazard models showed that high level of blood UCR were independent with the risk of CAD in patients living with T2DM (Table 3). After adjusting age, every 1.0 increase in one standardized unit of UCR was associated with a *1.257-fold (95%CI 1.044–1.513)* increased risk for CAD. After adjusting age, BMI, and DBP, every 1.0 increase in one standardized unit of UCR was associated with a *1.296-fold (95%CI 1.077–1.561)* increased risk for CAD. After adjusting age, BMI, DBP, HDL-C, eGFR, and TyG, every 1.0 increase in one standardized unit of UCR was associated with a *1.370-fold (95%CI 1.136–1.651)* increased risk for CAD. The 3 models’ results all showed that the multivariable-adjusted risk increased significantly with the increasing UCR levels. And higher level of blood UCR showed a higher risk of CAD significantly.

**Table3:** Associations between UCR levels and risk for CAD in patients living with T2DM

Variables	HR(95%CI) by UCR level		
	model1	model2	model3
UCR per 1 increment(n = 12,299)	1.257(1.044,1.513) ^*^	1.296(1.077,1.561) ^**^	1.370(1.136,1.651) ^**^
*Low_level (n* = *614)*	1(reference)	1(reference)	1(reference)
*Medium_level (n* = *11,067)*	1.070(0.801,1.431)	1.110(0.830,1.484)	1.190(0.889,1.592)
*High_level (n* = *618)*	1.487(1.037,2.133) ^*^	1.587(1.105,2.278) ^*^	1.782(1.237,2.567) ^*^
age	1.034(1.029,1.039) ^***^	1.038(1.033,1.044) ^***^	1.040(1.035,1.046) ^***^
Body Mass Index (BMI)	-	1.035(1.017,1.053) ^***^	1.021(1.003,1.040) ^*^
Diastolic Blood Pressure (DBP)	-	1.012(1.006,1.018) ^***^	1.011(1.005,1.016) ^***^
High-Density Lipoprotein Cholesterol (HDL-C)	-	-	0.705(0.553,0.900) ^**^
Estimated Glomerular Filtration Rate (eGFR)	-	-	1.003(1.001,1.005) ^**^
Triglyceride glucose (TyG)	-	-	1.130(1.038,1.231) ^**^
C-statistics (95% CI)	0.630(0.621,0.638)	0.635(0.627,0.644)	0.644(0.636,0.652)

^*^P < 0.05; **P < 0.01, ***P < 0.001

*HR* hazard ratio, *CI* confidence interval for hazard ratio of UCR

Cut-off values of UCR: Low_level, ≤ 4.36; Medium_level, (4.36,10.45]; High_level,  > 10.45

### Nonlinear association between UCR and risk of CAD

**Fig. 4:**
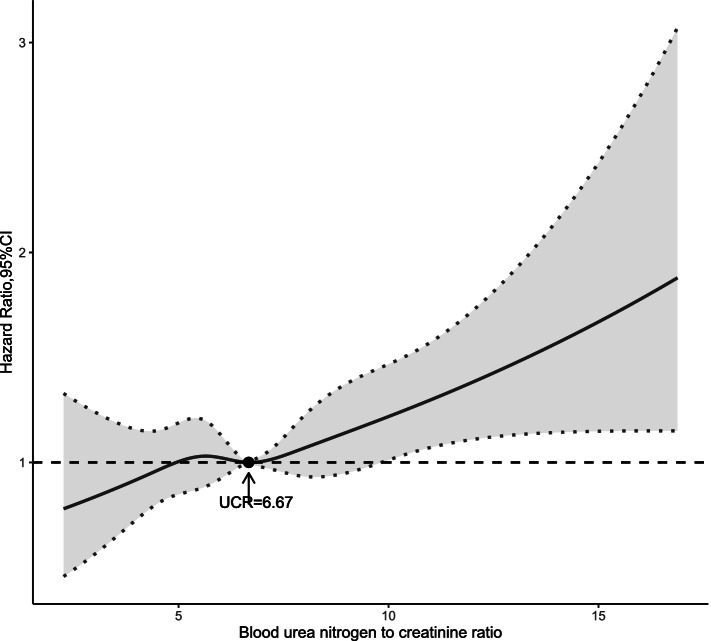
Restricted cubic spline showing the adjusted association between UCR and incidence of CAD

Multivariable restricted cubic spline function for CAD is presented in Fig. 4. After adjusting all potential confounders mentioned above, restricted cubic spline showed a curved association between UCR levels and risk of CAD in patients living with T2DM. Cubic spline function graph suggested that the influence of UCR level on HR for CAD increased significantly at UCR levels above 6.67.

## Discussion

The increasing global prevalence of T2DM and chronic kidney disease (CKD) has prompted research efforts to tackle the growing epidemic of diabetic kidney disease (DKD; also known as diabetic nephropathy) [30]. Daniela Dunkler et al. proved that eGFR were the most important factors to predict onset and progression of early CKD in individuals with type 2 diabetes [31]. Haijing Jiang et al. found that a mild-to-severe decline in eGFR or a raised level of BUN might be associated with increased risk of incident CAD in middle-aged and elderly Chinese populations [13]. Furthermore, the blood urea nitrogen has emerged as an independent predictor of poor clinical outcomes in various population settings. However, epidemiological evidence that related UCR to risk of incident CAD in patients living with T2DM was still limited.

On the basis of a large cohort of health management Chinese populations, we reported that blood UCR is the independent risk factor for CAD risk in T2DM patients. After adjusting age, BMI, DBP, HDL-C, eGFR, and TyG, every 1.0 increase in one standardized unit of UCR was associated with a *1.370-fold (95%CI 1.136–1.651)* increased risk for CAD. The results suggest that the new biomarker-blood UCR- should be considered in the risk prediction and prevention of CAD among patients living with T2DM. From the perspective of pathophysiology, the increase of UCR indicates that body metabolic decomposition is strengthened, which will lead to the decrease of muscle mass. There is a certain correlation between the decrease of muscle mass and T2DM. The possible mechanism is that people with T2DM are in a state of chronic systemic inflammation, which leads to impaired glucose regulation of skeletal muscle, and further causes a decrease in muscle tissue and an increase in adipose tissue. The reduction of muscle tissue can reduce the glycogen storage function of skeletal muscle, which will make the excess blood sugar in the body unable to be converted into glycogen through insulin, and increase insulin resistance in turn. Increasing insulin resistance of adipose tissue will lead to the deposition of large amounts of free fatty acids (FFA) which distribute in liver and peripheral tissues, hinder the oxidation and transport of glucose, and cause abnormal blood lipid metabolism. These changes may increase the risk of Coronary Artery Disease (CAD) [32].

On the basis of a large cohort of health management Chinese populations, we reported that an elevated blood UCR level was associated with a higher risk of CAD in patients living with T2DM independently of traditional cardiovascular risk factors (such as BMI, blood pressure, blood sugar, eGFR, insulin resistance, and so on). Our findings added to the value of epidemiological evidence that current biomarker of renal function is associated with CAD risk in T2DM patients, suggesting that the new biomarker-blood UCR- should be considered in the risk prediction and prevention of CAD among patients living with T2DM.

The following limitations should be considered in our study. First, we did not have exhaustive details about the possible causes of evaluated blood UCR in patients living with T2DM, such as increased calorie intake. Second, although many factors are affecting CAD, this study can only adjust for limited confounding due to limited data. Third, the role of muscle mass should be analysed when dealing with creatinine, although through checking the data in the database, it is found that only a few participants have a record of muscle mass, and it is impossible to analyse the impact of muscle mass on research. Forth, since we can’t obtain information about the severity of CAD from health management data, it is impossible to analyse the correlation between UCR levels and the severity of CAD in patients living with T2DM. Fifth, as there are no references for outlier ranges of the indicators for the health management population, we have to use the outlier ranges of the diseased population indicators defined by authoritative epidemiologist to replace.

## Conclusions

Our study demonstrates that UCR is an independent risk factor for CAD in patients living with new-onset T2DM. Moreover, the results suggested that the influence of UCR level on HR for CAD increased significantly at UCR levels above 6.67. According to our study, an elevated UCR can increase the risk of CAD in patients living with T2DM. This study provides a theoretical basis for tertiary prevention in the T2DM population.

## Data Availability

The datasets used and/or analysed during the current study are available from the corresponding author on reasonable request.

## Abbreviations

DM: Diabetes Mellitus
IDF: International Diabetes Federation
T2DM: Type 2 Diabetes Mellitus
CAD: Coronary Artery Disease
BUN: Blood Urea Nitrogen
SCr: Serum Creatinine
UCR: Blood Urea Nitrogen to Creatinine Ratio
WBC: White Blood Cell Count
NEUT: Neutrophil Count
LYM: Lymphocyte Count
FPG: Fasting Plasma Glucose
FPG_F: Fasting Plasma Glucose Followed
TC: Total Cholesterol
TG: Triglyceride
HDL-C: High-Density Lipoprotein Cholesterol
LDL-C: Low-Density Lipoprotein Cholesterol
BMI: Body Mass Index
WHR: Waist-to-Hip Ratio
SBP: Systolic Blood Pressure
DBP: Diastolic Blood Pressure
WHO: World Health Organization
HbA1c: Glycosylated Haemoglobin A1c
UA: Uric Acid; ECG: Electrocardiographic
CKD: Chronic Kidney Disease; DKD: Diabetic Kidney Disease
